# BMC Nephrology reviewer acknowledgement 2012

**DOI:** 10.1186/1471-2369-14-61

**Published:** 2013-04-02

**Authors:** Hayley Henderson

**Affiliations:** 1 BioMed Central, Floor 6, 236 Gray's Inn Road, London WC1X 8HB, United Kingdom

## Abstract

**Contributing reviewers:**

The editors of BMC Nephrology would like to thank all our reviewers who have contributed to the journal in Volume 13 (2012).

## 

Kevin Abbott

USA

Teresa Adragao

Portugal

Mayank Agarwal

India

Sudhanshu Agrawal

USA

Varun Agrawal

USA

Joseph Ahearn

USA

Curie Ahn

Korea South

Ipek Akil

Turkey

Guerin Alain

France

Ziyad Al-Aly

USA

Mohamed Farouk Allam

Egypt

Gabriel Almroth

Sweden

Dante Amato

Mexico

Csaba Ambrus

Hungary

John Anderson

USA

Minoru Ando

Japan

Ryoichi Ando

Japan

Simeone Andrulli

Italy

Bilgin Kadri Aribas

Turkey

Mustafa Arici

Turkey

Margaret Ashcroft

United Kingdom

Shervin Assari

Iran

Bjørn Åsvold

Norway

Daniel Athanazio

Brazil

Harold Aukema

Canada

Alper Azak

Turkey

Justine Bacchetta

France

Debasish Banerjee

United Kingdom

Heejung Bang

USA

Kamil Barbour

USA

Roberto José Barone

Argentina

Fellype Barreto

Brazil

Bahar Bastani

USA

Judy Bauer

Australia

George Bayliss

USA

Ilia Beberashvili

Israel

Antonio Bellasi

Italy

Carsten Bergmann

Germany

Raoul Bergner

Germany

Harald Bergrem

Norway

Anatole Besarab

USA

Michiel Betjes

Netherlands

Ishir Bhan

USA

Sunil Bhandari

United Kingdom

Rajendra Bhimma

South Africa

Jean-Jacques Boffa

France

C Böger

Germany

Carsten Böger

Germany

Piergiorgio Bolasco

Italy

Thomas Bollen

Netherlands

Kyra Borchhardt

Austria

Neil Boudville

Australia

Edward Bourry

France

Simon Bowman

United Kingdom

Jennifer Bragg-Gresham

USA

Vincent Brandenburg

Germany

Tobias Breidthardt

Switzerland

Marie-Luise Brezniceanu

Canada

David Brieger

Australia

Sergey Brodsky

USA

Steven Brunelli

USA

Nikolay Bukanov

USA

Ludmila Buravkova

Russian Federation

Stephane Burtey

France

Jadranka Buturovic-Ponikvar

Slovenia

Paolo Calzavacca

Italy

Josep Campistol

Spain

Ahmet Selçuk Can

Turkey

Bernard Canaud

France

Bernard Canaud

France

Changchun Cao

China

Timothy Card

United Kingdom

Alan Cass

Australia

Giuseppe Castellano

Italy

Pietro Castellino

Italy

Filomena Cetani

Italy

Anthony Chang

USA

Ming-Yang Chang

Taiwan

Christos Chatzikyrkou

Germany

Kunal Chaudhary

USA

Charles Chazot

France

Ching-Feng Cheng

Taiwan

Stephanie Cherqui

USA

Sau Cheung

USA

Ho Jun Chin

Korea South

Yi-Wen Chiu

Tanzania

Archil Chkhotua

Georgia

Bum Soon Choi

Korea South

Murim Choi

USA

Tzong-Shinn Chu

Taiwan

Chuhan Chung

USA

Patricia Cleary

USA

Aldo Clerico

Italy

Mary Petrea Cober

USA

Steven Coca

USA

Clemens Cohen

Switzerland

Michael Copland

Canada

Benilde Cosmi

Italy

Elísio Costa

Portugal

Cécile Couchoud

France

Adrian Covic

Romania

Richard Coward

United Kingdom

Daniel Coyne

USA

Emanuele Cozzani

Italy

Paolo Cravedi

Italy

Wenpeng Cui

China

Jonathan Cullis

United Kingdom

Jan Danser

Netherlands

Marcio Dantas

Brazil

Subir Kumar Das

India

Indranil Dasgupta

United Kingdom

Angel Lm De Francisco

Spain

Carlos Abaete De Los Santos

Brazil

Andreana De Mauri

Italy

Johan De Meester

Belgium

Luca De Nicola

Italy

Sacha De Serres

Canada

Renato De Vecchis

Italy

Jan De Waele

Belgium

John Dean

United Kingdom

Serpil Muge Deger

Turkey

Lucia Del Vecchio

Italy

Pierre Delanaye

Belgium

Yahsou Delmas

France

Vimal Derebail

USA

Joep Derikx

Netherlands

Patrick D'Haese

Belgium

Matteo Nicola Dario Di Minno

Italy

Kent Doi

Japan

Carlo Donadio

Italy

Adriana Dusso

Spain

Adriana Dusso

Spain

Tevfik Ecder

Turkey

Aydin Ece

Turkey

Alberto Edefonti

Italy

Somchai Eiam-Ong

Thailand

Robert Ekart

Slovenia

Khalid Elased

USA

Ahmed El-Assmy

Egypt

Theodoros Eleftheriadis

Greece

Rosilene Elias

Brazil

Manal Elshamaa

Egypt

Francesco Emma

Italy

Philipp Enghard

Germany

Giuseppe Enia

Italy

Ricardo Enríquez

Spain

Vera Eremina

Canada

F. Fevzi Ersoy

Turkey

Pasquale Esposito

Italy

Vittoria Esposito

Italy

Michelle Estrella

USA

Joseph Eustace

Ireland

Tevfik Rifki Evrenkaya

Turkey

Fabio Fabbian

Italy

Stephen Fadem

USA

Jeffrey Fadrowski

USA

Fadi Fakhouri

France

Stanley Fan

United Kingdom

Robert Fassett

Australia

Ben Moussa Fatma

Tunisia

Sarah Faubel

USA

Mariano Feriani

Italy

Giuseppe Ferrante

Italy

Inês Ferreira

Portugal

Sebastiao Ferreira-Filho

Brazil

Vassilis Filiopoulos

Greece

Guido Filler

Canada

Guido Finazzi

Italy

Steven Fishbane

USA

Michael Flessner

USA

Oliver Flossmann

United Kingdom

Costas Fourtounas

Greece

Andrew Frankel

United Kingdom

Ping Fu

China

Michiaki Fukui

Japan

Luciana Furci

Italy

Maria Fusaro

Italy

Carlo Gaillard

Netherlands

Andrea Galassi

Italy

Kresimir Galesic

Croatia

Hugh Gallagher

United Kingdom

Frances Game

United Kingdom

Santiago Garcia

USA

Guillermo Garcia Garcia

Mexico

Adam Gaweda

USA

Simonetta Genovesi

Italy

Sandra Giannelli

Switzerland

Antonietta Gigante

Italy

Maddalena Gigante

Italy

David Gilbertson

USA

Justyna Gołębiewska

Poland

Arda Goceroglu

Netherlands

Gabriela Godaly

Sweden

David Goldfarb

USA

Alexander Goldfarb-Rumyantzev

USA

Stuart Goldstein

USA

Emilio Gonzalez-Parra

Spain

William Goodman

USA

Louisa Gordon

Australia

Jose L Gorriz

Spain

David Gracey

Australia

Morgan Grams

USA

Eirini Grapsa

Greece

Olaf Grisk

Germany

Fabrizio Grosjean

Italy

Jakob Gubensek

Slovenia

Ashima Gulati

USA

Richard Gun

Australia

Samir Gupta

USA

Orlando Gutierrez

USA

Michael Haase

Germany

Anja Haase-Fielitz

Germany

Mehryar Habibi Roudkenar

Iran

Tadeusz Halatek

Poland

Seung Hyeok Han

Korea South

Kevin Harris

United Kingdom

Hans Hartmann

Germany

Jean-Philippe Haymann

France

Ron Hays

USA

James Heaf

Denmark

Manfred Hecking

Austria

Martinez Ramirez Hector Ramon

Mexico

Saskia Heeringa

Switzerland

Lambers Heerspink

Netherlands

Charles Heilig

USA

Gunnar Heine

Germany

Leal Herlitz

USA

Kouichi Hirayama

Japan

Swapnil Hiremath

Canada

Elisabeth Hodson

Australia

Hallvard Holdaas

Norway

Kristina Hölig

Germany

Jianghui Hou

USA

Martin Howell

Australia

Yu-Juei Hsu

Taiwan

Zhiying Huang

China

Ender Hur

Turkey

Alastair Hutchison

United Kingdom

Shang-Jyh Hwang

Taiwan

Melissa Hyde

Australia

Alma Idrizi

Albania

Ryota Ikee

Japan

Yohei Ikezumi

Japan

Tadaatsu Imaizumi

Japan

Atiporn Ingsathit

Thailand

Lesley Inker

USA

Jula Inrig

USA

Joji Ishikawa

Japan

Liotier Jerome

French Polynesia

Bernard Jaar

USA

Ralf Jacob

Germany

Kitty Jager

Netherlands

Diana Jalal

USA

Joachim Jankowski

Germany

Paul Jennings

Austria

Anita Jeyakumar

USA

Ratan Jha

India

Kenar Jhaveri

USA

Rahul Jindal

USA

Lars Jødal

Denmark

Kirsten Johansen

USA

Rachel Johnson

United Kingdom

Stacey Jolly

USA

Daryl Andrew Jones

Australia

Graham Jones

Australia

Sofia Jorge

Portugal

Noemie Jourde-Chiche

France

Noemie Jourde-Chiche

France

Claudine Jurkovitz

USA

Shyh-Chuan Jwo

Taiwan

Kamyar Kalantar-Zadeh

USA

Yuji Kamijo

Japan

Christoph Kampmann

Germany

Phillip Kantharidis

Australia

Fiona Karet

United Kingdom

Constantine Karvellas

Canada

Belde Kasap

Turkey

Frederick Kaskel

USA

Efstathios Kastritis

Greece

Pranay Kathuria

USA

Akihiko Kato

Japan

Johji Kato

Japan

Mansur Kayata

Turkey

Kubra Kaynar

Turkey

Mira Keddis

USA

Andre P Kengne

Cameroon

David Kershaw

USA

Gheun-Ho Kim

Korea South

Elzbieta Kimak

Poland

Dimitrios Kirmizis

Greece

Andreas Kistler

Switzerland

Chagriya Kitiyakara

Thailand

David Kluth

United Kingdom

Cheol Woo Ko

Korea South

Mehmet Koc

Turkey

Gülay Koçak

Turkey

Ismail Kocyigit

Turkey

Masahiro Kohzuki

Japan

Javad Kojuri

Iran

Hirotaka Komaba

Japan

Tsuneo Konta

Japan

Ron Korstanje

USA

Michael Kostapanos

Greece

Efstathios Koulouridis

Greece

Susan Kralisch-Jäcklein

Germany

Andrea Kramer

Netherlands

Lissy Krishnan

India

Thierry Krummel

France

Lauren Kucirka

USA

Satoru Kuriyama

Japan

Daniel Kurnik

Israel

Tetsuro Kusaba

USA

Tae-Hwan Kwon

Korea South

Gaetano La Manna

Italy

Olivier Lada

France

Edmund Lamb

United Kingdom

Daniel Landau

Israel

Brian Lane

USA

Nicola Latronico

Italy

Chiara Lazzeri

Italy

Vincent Lee

Australia

Kevin Lemley

USA

Annette Lennerling

Sweden

Adeera Levin

Canada

Visnja Lezaic

Serbia

Karl Lhotta

Austria

Orfeas Liangos

Germany

Tomasz Liberek

Poland

Alexandre Liborio

Brazil

John Lieske

USA

Wai Lim

Australia

Chih-Ching Lin

Taiwan

Shih-Hua Lin

Taiwan

Marshall D Lindheimer

USA

Bengt Lindholm

Sweden

Gabor Egon Linthorst

Netherlands

Jiannong Liu

USA

Francesco Locatelli

Italy

Jose Antonio Lopes

Portugal

Francisco Lopez-Hernandez

Spain

Maria-Angela Losi

Italy

Jiing-Chyuan Luo

Taiwan

Antonio Lupo

Italy

Yingchun Ma

China

Mark Macgregor

United Kingdom

Richard Macisaac

Australia

Francois Madore

Canada

Denise Mafra

Brazil

John Mahan

USA

Bakhtawar Mahmoodi

Netherlands

Marta Makara-Studziska

Poland

Yuichi Makino

Japan

Ewa Malecka-Tendera

Poland

Leila Malekmakan

Iran

Deepak Malhotra

USA

Pavlos Malindretos

Greece

Francesca Mallamaci

Italy

Roberto Manfredini

Italy

Nseka Mangani

Congo Democratic Republic

Johannes Mann

Germany

M. Loredana Marcovecchio

Italy

Christophe Mariat

France

Patrick Mark

United Kingdom

Julia Marley

Australia

David Marples

United Kingdom

Mark Marshall

New Zealand

Lih-Wen Mau

USA

Evangelos Mazaris

United Kingdom

William Mcclellan

USA

Aj Mcknight

United Kingdom

Lawrence Mcmahon

Australia

Esther Meijer

Netherlands

Bjorn Meijers

Belgium

Nikolaos Melas

Greece

Jose Méndez

Mexico

Wieneke Michels

USA

Akira Mima

Japan

Roberto Minutolo

Italy

Gabriel Mircescu

Romania

Koji Mita

Japan

Anna Paola Mitterhofer

Italy

Sumit Mohan

USA

Masoumeh Mohkam

Iran

Miklos Zsolt Molnar

Hungary

Karen Molyneux

United Kingdom

Santo Morabito

Italy

Matthew Morgan

United Kingdom

Yasukiyo Mori

Japan

Tatsumi Moriya

Japan

Takahito Moriyama

Japan

Mohamed Morsy

United Kingdom

Rosa Maria Moyses

Brazil

Muhammed Mubarak

Pakistan

Rebecca Muehrer

USA

Giuseppe Mule'

Italy

Mariana Murea

USA

Fliss Murtagh

United Kingdom

Ahmet Yaser Muslumanoglu

Turkey

Thangamani Muthukumar

USA

N.S. Patel

United Kingdom

Tibor Nadasdy

USA

Shizuko Nagao

Japan

Yasuyuki Nagasawa

Japan

Saraladevi Naicker

South Africa

Koshi Nakamura

Japan

Koichi Nakanishi

Japan

Hitoshi Nakashima

Japan

Manouchehr Nakhjavani

Iran

Serafeim Nanas

Greece

Joseph Naoum

Lebanon

Andrew Narva

USA

Sankar Navaneethan

USA

Juan Navarro-González

Spain

Armando Negri

Argentina

Edward Nemeth

Canada

Kenneth Nepple

USA

James Neuberger

United Kingdom

Tryggve Nevéus

Sweden

Britt Newsome

USA

Susanne Nicholas

USA

Thomas Nickolas

USA

Gian Luigi Nicolosi

Italy

Zofia Niemir

Poland

Monika Niewczas

USA

Hiroshi Nishi

USA

Ravi Nistala

USA

Kosaku Nitta

Japan

Wenquan Niu

China

Alessandro Nobili

Italy

Helen Noble

United Kingdom

Silas P Norman

USA

Yoshitsugu Obi

Japan

Tetsuya Ogawa

Japan

Naro Ohashi

Japan

Isao Ohsawa

Japan

Takayasu Ohtake

Japan

Rieko Okada

Japan

Ikechi Okpechi

South Africa

Bola Ola

Nigeria

João Paulo Oliveira

Portugal

Ali Mirza Onder

USA

Albert Ong

United Kingdom

Luiz Onuchic

Brazil

Macaulay Onuigbo

USA

Lori Orlando

USA

Paula Ormandy

United Kingdom

Francisco Ortega

Spain

Pablo A Ortiz

USA

Shahrzad Ossareh

Iran

Marlies Ostermann

United Kingdom

John O'Toole

USA

Andrea Paiva

USA

Amir Pakpour

Iran

Alberto Palazzuoli

Italy

Nicolas Pallet

France

Aikaterini Papagianni

Greece

Nisha Parikh

USA

Mark Parker

USA

Samir Patel

USA

Tejas Patel

USA

Sandipan Pati

USA

Robert Pauly

Canada

Krystyna Pawlak

Poland

York Pei

Canada

Yu-Sen Peng

Taiwan

Lars Penne

Netherlands

Kristina Penniston

USA

Jazmin Perez

Mexico

Frederik Persson

Denmark

Manuel Pestana

Portugal

John Pickering

New Zealand

Chrysoula Pipili

Greece

Laura Plantinga

USA

Roberta Poletti

Italy

Juan Politei

Argentina

Cesare Polito

Italy

Kevan Polkinghorne

Australia

Roberto Pontremoli

Italy

Hans Pottel

Belgium

Albert Power

United Kingdom

Kearkiat Praditpornsilpa

Thailand

Minolfa Prieto

USA

Patrick Pun

USA

José Ramón Quevedo

Spain

Richard Quigg

USA

Giuseppe Quintaliani

Italy

Ram R

India

Soroush Rais-Bahrami

USA

Meera Ramanujam

USA

Ganesan Ramesh

USA

Manuel Ramos-Casals

Spain

Senija Rasic

Bosnia and Herzegovina

Knud Rasmussen

Denmark

Manish Rathi

India

Pablo Rebollo

Spain

Stephan Reinhold

Germany

Flavio Reis

Portugal

Jochen Reiser

USA

Scott Reisman

USA

Hanna Rennert

USA

Stephen Riley

United Kingdom

Antonio Rios

Spain

Bengt Rippe

Sweden

Michael Robson

United Kingdom

James Rodrigue

USA

Rudolph Rodriguez

USA

Steven Rosansky

USA

Alexander Rosenkranz

Austria

Sandro Rossetti

USA

Guy Rostoker

France

Paula Rozenfeld

Argentina

Romana Rysava

Czech Republic

Dong-Ryeol Ryu

Korea South

Alaa Sabry

Egypt

Milenka Sain

Croatia

Ramin Sam

USA

Marcelo Santos Sampaio Sampaio

Brazil

Pantelis Sarafidis

United Kingdom

Yoshie Sasatomi

Japan

Patrick Saudan

Switzerland

Vincenzo Savica

Italy

Francesco Paolo Schena

Italy

Felix Jv Schlosser

USA

Holger Schmid

Germany

Johannes Schoedel

Germany

Marianne Schoorl

Netherlands

Doron Schwartz

Israel

Julia Scialla

USA

Yoav Segal

USA

Alfonso Segarra

Spain

Julian Segura

Spain

Nicholas Selby

United Kingdom

Govindan Sadasivam Selvam

India

Eun Young Seong

Korea South

Vincenzo Sepe

Italy

Vincenzo Sepe

Italy

Andreas Serra

Switzerland

Mary Ann Sevick

USA

Meltem Sezis Demirci

Turkey

Tariq Shafi

USA

Faissal Shaheen

Saudi Arabia

Vahakn Shahinian

USA

Eduard Shantsila

United Kingdom

Mukut Sharma

USA

Min Shi

USA

Chih-Chung Shiao

Taiwan

Ho Sik Shin

Korea South

Theresa Shireman

USA

Tetsuo Shoji

Japan

Muhammed Siddiqui

United Kingdom

Marcia Silver

USA

James Simon

USA

Jan Simoni

USA

Ian Simpson

New Zealand

Juha Sinisalo

Finland

Ekaterini Siomou

Greece

Christine Skerka

Germany

Fotini Skopouli

Greece

Yelena Slinin

USA

Roy L. Soiza

United Kingdom

María José Soler Romeo

Spain

Jun Soma

Japan South

Young Rim Song

Korea

Manish Sood

Canada

Stephen Sozio

USA

Walter Speidl

Austria

Nattachai Srisawat

USA

Giovanni Stallone

Italy

Maria Stangou

Greece

Aristeidis Stavroulopoulos

Greece

Cj Stefanidis

Greece

Peter Stenvinkel

Sweden

Andréa Stinghen

Brazil

Tomasz Stompor

Poland

Yves St-Pierre

Canada

Pawel Strozycki

Poland

Kostas Stylianou

Greece

Toshihiro Sugiura

Japan

Hitoshi Sugiyama

Japan

Jennfier Sullivan

USA

Ernest Sumaili

Congo Democratic Republic

Keiichi Sumida

Japan

Manish Suneja

USA

Junne-Ming Sung

Taiwan

Andrzej Surdacki

Poland

Kalathil Sureshkumar

USA

Eva Swahn

Sweden

Pawel Szulc

France

Silke Szymczak

USA

Maarten Taal

United Kingdom

Yoshifumi Tada

Japan

Takamune Takahashi

USA

Ayumi Takakura

USA

Girish Talaulikar

Australia

Ying-Cai Tan

USA

Navdeep Tangri

Canada

Erhan Tatar

Turkey

Silvana Tedeschi

Italy

Boon Wee Teo

Singapore

Vladimir Tesar

Czech Republic

Frank Thevenod

Germany

Nathalie Thilly

France

Micah Thorp

USA

Micah Thorp

USA

Raj Thuraisingham

United Kingdom

Francesca Tinti

Italy

Andreas Tomaschitz

Austria

Anna Tomaszuk-Kazberuk

Poland

Okada Tomonari

Japan

Burkhard Tönshoff

Germany

Marten Trendelenburg

Switzerland

Charikleia Triantopoulou

Greece

Nicola Troisi

Italy

Joan Carles Trullas

Spain

Vasilis Tsimihodimos

Greece

Nobuo Tsuboi

Japan

Hiroyasu Tsukaguchi

Japan

Delphine Tuot

USA

Faruk Turgut

Turkey

Kultigin Turkmen

Turkey

Shinichi Uchida

Japan

Andreea Udrea

Romania

Andrew Udy

Australia

Katrin Uhlig

USA

Michael Ullian

USA

Aydin Unal

Turkey

Mark Unruh

USA

Pablo Antonio Urena Torres

France

Boris Utsch

Germany

Prayong Vachvanichsanong

Thailand

Nicole Van De Kar

Netherlands

Maria Van Den Muijsenbergh

Netherlands

Gijs Van Pottelbergh

Belgium

Ivana Vaneckova

Czech Republic

Kriengsak Vareesangthip

Thailand

Nikhil Vasdev

United Kingdom

Joseph Vassalotti

USA

Joachim Velden

Germany

Ivan Velez

Colombia

Rocco Venuto

USA

Humberto Villacorta

Brazil

Emmanuel Villar

France

Libor Vitek

Czech Republic

Katherine Wachmann

USA

Hani Wadei

USA

Rob Walker

New Zealand

Haiyan Wang

China

Jiguang Wang

China

Weiming Wang

China

Steven Weisbord

USA

Donald Wesson

USA

Sarah White

USA

Natasha Wiebe

Canada

Niels Wiinberg

Denmark

Benjamin Wilde

Netherlands

Andrew Williams

Australia

Jay Wish

Vanuatu

Germaine Wong

Australia

Markus Wörnle

Germany

Mai-Szu Wu

Taiwan

Rudolf P. Wüthrich

Switzerland

Li Xiangpei

China

Peng-Cheng Xu

China

Kunihiro Yamagata

Japan

Yucheng Yan

China

Motoko Yanagita

Japan

Baoxue Yang

China

Hui Kim Yap

Singapore

Halil Yazici

Turkey

Ajay Yerramasu

United Kingdom

Tae-Hyun Yoo

Korea South

Peter Yuen

USA

Nadia Zalunardo

Canada

Zaki Anas Zarka

Syria

Luxia Zhang

China

Minghui Zhao

China

Fude Zhou

China

Fansan Zhu

USA

Gaston Zilleruelo

USA

Anna Zisman

USA

Emanuel Zitt

Austria

Carmine Zoccali

Italy

## Pre-publication history

The pre-publication history for this paper can be accessed here:

http://www.biomedcentral.com/1471-2369/14/61/prepub

